# Structure-dependent mechanical properties of ultrathin zinc oxide nanowires

**DOI:** 10.1186/1556-276X-6-352

**Published:** 2011-04-20

**Authors:** Wen-Jay Lee, Jee-Gong Chang, Shin-Pon Ju, Meng-Hsiung Weng, Chia-Hung Lee

**Affiliations:** 1 National Center for High-Performance Computing, No. 28, Nan-Ke Third Road, Hsin-Shi, Tainan 74147, Taiwan; 2 Department of Mechanical and Electro-Mechanical Engineering, Center for Nanoscience and Nanotechnology, National Sun Yat-sen University Kaohsiung, 804, Taiwan

## Abstract

Mechanical properties of ultrathin zinc oxide (ZnO) nanowires of about 0.7-1.1 nm width and in the unbuckled wurtzite (WZ) phase have been carried out by molecular dynamics simulation. As the width of the nanowire decreases, Young's modulus, stress-strain behavior, and yielding stress all increase. In addition, the yielding strength and Young's modulus of Type III are much lower than the other two types, because Type I and II have prominent edges on the cross-section of the nanowire. Due to the flexibility of the Zn-O bond, the phase transformation from an unbuckled WZ phase to a buckled WZ is observed under the tensile process, and this behavior is reversible. Moreover, one- and two-atom-wide chains can be observed before the ZnO nanowires rupture. These results indicate that the ultrathin nanowire possesses very high malleability.

## Introduction

Wide band gap semiconductor materials, such as AlN, GaN, and ZnO, have attracted a lot of attention in the past because of their excellent performance in electronic, optoelectronic, and piezoelectric properties [1,2]. As for the II-VI semiconductor material compound ZnO, it possesses a wide direct band gap (3.37 eV) and a strong excitation binding energy (60 MeV), such that it can be used in solar cells [3-7], optical sensitizers [8], and quantum devices. In 2001, Feick et al. identified one-dimensional ZnO nanorods [9], and since then many experts have successively identified and synthesized various kinds of ZnO nanostructures by experiment [1,2,10-18]. Manoharan [19] synthesized ZnO nanowires with diameters of 200-750 nm by using the vapor-liquid-solid (VLS) technique.

In 2006, Wang et al. [1,2] found that the ZnO nanowire has piezoelectric property which can convert nanoscale energies such as mechanical, vibrational, or hydraulic energy into electrical energy in bending deformation. This performance indicates that the ZnO nanowire has both semiconducting and piezoelectric properties. This result allows the ZnO nanowire to have some applications in energy output by material deformation. He et al. [15] have performed nanomanipulation to measure the *in situ I-V *characteristics of a single ZnO nanowire. It has been demonstrated that a single ZnO nanowire can be a rectifier simply by mechanically bending it, similar to a *p-n *junction-based diode. Utilizing the coupled piezoelectric and semiconducting dual properties of ZnO, ZnO nanowire was used to compose piezoelectric field-effect transistors (PE-FET), which was then demonstrated as a force sensor in the nanonewton range [20]. Fei et al. [21] report that the bent ZnO PFW cantilever can create a piezoelectric potential distribution across its width at its root and simultaneously produce a local reverse depletion layer with a much higher donor concentration than normal, dramatically changing the current flowing from the source electrode to drain electrode when the device is under a fixed voltage bias.

Because of the excellent properties and diversified application of the ZnO nanowire, it is necessary to develop a precise understanding of the mechanical property of ZnO nanowire. Experimentally, Manoharan [19] measured the Young's modulus of the ZnO nanowires with diameters of 200-750 nm by performing cantilever bending experiments and found that the Young's modulus was estimated to be about 40 GPa, which is smaller than that in the bulk scale (140 GPa). However, Chen et al. [22] and Agrawal et al. [23] found that the Young's moduli increased as the diameter decreased, and the values of these Young's moduli were larger than the bulk value. On the theoretical side, Kulkarni et al. [24] used molecular dynamics (MD) and first principles calculations to investigate the phase transformation of ZnO nanowires from wurtzite (WZ) to a graphite-like hexagonal (HX) structure under uniaxial tensile strain. Their results show that the WZ and HX structures of ZnO nanowires had different properties. The stress-induced phase transformation significantly alters the modulation of piezoelectric constant and thermal conductivity of the nanowire. Wang et al. [25] also reported structural transformation from WZ to HX structure for ultrathin ZnO nanowires under uniaxial elongation and compression. Moreover, the band gap, Young's modulus, and Milliken charges were calculated by density functional theory (DFT), and it was found that the band gaps of ZnO nanowires depended on the size and geometry of the nanowire, i.e., the WZ phase of ZnO nanowires had a larger band gap than the HX phase. However, for Young's modulus of the ZnO nanowires, HX phase was higher than the WZ phase, increasing with a decrease in the size of ZnO naowires (HX). Wu et al. [26] found a structure transformation by DFT calculation from the HX phase to WZ phase as the diameter and length of the AlN, GaN and ZnO nanowire increased. They also justified that the phase transformation is caused by competition between the bond energy, the Coulomb energy, and the energy originating from the dipole field of the WZ structure. Hu et al. [27] and Wang et al. [25] presented the mechanical properties of ZnO nanowires with WZ structure and nanotubes as a function of diameter by using MD simulation. Their results show that Young's modulus of ZnO nanowire is inversely proportional to the diameter of nanowire, which is a result in agreement with Wang et al. [20]. They demonstrate that the size-dependent elastic properties of nanowires principally arise from the stress-induced surface stiffening. Wang et al. [28] found a novel stress-strain relationship with two stages of linear elastic deformation in [0001]-oriented ZnO nanorods under tensile loading [28]. This phenomenon is caused by a phase transformation from WZ to a body-centered tetragonal structure with four-atom rings (BCT-4). In addition, they show that the two stages of linear elastic deformation still exist at a high temperature of 1500 K. Dai et al. [29] found the single atom chain structure during the tensile process. They explain that the growth of the single atom chain results from the bond breakage at the junction of the chain and the amorphous bulk. Moreover, they also propose a mechanics-based criterion for neck propagation. However, the Young's modulus and yielding stress of ZnO nanowires with thickness less than 1.6 nm is much lower than the other larger cases, which could be due to the significant deformation in the initial structure.

Until now, the research of the mechanical properties of ZnO nanowires with HX structure has concentrated almost solely on the elastic property. There is still no research discussing the deformation mechanism in detail. As a result, the present work uses MD simulation for detailed discussions of the mechanical properties (yield stress and Young's modulus) and the deformation behavior of ZnO nanowires under uniaxial tension.

## Simulation model

In the present study, the mechanical property and deformation behavior of ZnO nanowires in the HX phase are investigated by MD simulations [30,31]. To understand the lateral size effect of HX phase ZnO nanowires, three ZnO nanowires were chosen as the initial systems. These nanowires were initially in the WZ phase, and had a diameter of about 0.7-1.1 nm and length of 7.2 nm, because only ZnO nanowires with diameters less than 1.3 nm can transform to the HX phase [25,26]. After the structural optimization by Genetic Algorithms software module, the ZnO nanowires transform to the HX phase. For the tensile test, canonical ensemble (NVT ensemble) [30,31] is employed in the MD simulation. We intercept in the middle region of the optimized nanowires, because the structure of both ends is somewhat nucleated, which could affect the intrinsic property of the nanowire. The details will be discussed in the first paragraph of "Results and discussion" section. The lengths of three nanowires are all set 5.5 nm. The atom numbers for the three nanowires are 364, 448, and 512. Prior to elongation, the Zn atoms and O atoms consist of two atomic layers at both ends of the ZnO nanowire, which are kept fixed, whereas the remaining layers are the thermal control portion. This relaxation process was used to eliminate the internal stresses. For the thermal control portion, the Nosè-Hoover method is adopted to ensure a constant system temperature at 1 K throughout the elongation procedure and the Velocity Verlet algorithm [30,31] is also employed to calculate the trajectories of the atoms. A time step of 1 fs was set for the time integration. In the axial tensile process, a tension with strain rate 0.02% ps^-1 ^is applied to the nanowire by applying a constant velocity to the two fixed layers in the axial direction. To measure the stress of the ZnO nanowire under elongation, the formulation of atomic level stress [32] is employed, which includes the kinetic and potential effects.

In the present study, the Buckingham potential of short-range interaction and Coulomb-electrostatic potential of long-range interaction are combined as the interatomic potential to simulate the interaction between the atoms of the ZnO nanowire, which is shown in Equation 1 as follows:(1)

where *A*_ij_, ρ_ij_, and *c*_ij _in Equation 1 are variable parameters given in reference [33]. *r*_ij _is the distance between i and j atoms. The first term in Equation 1 represents the long-range Coulomb interactions between two ions, and the second and third terms describe the overlap repulsion between atoms and the dipole-dipole interaction. The Ewald summation is employed to increase the computational efficiency in calculations of the long-range interaction of Coulomb force. In addition, the Coulomb interactions are usually modeled by constant charges for different ions. Such a model limits the possibility to describe a charge redistribution at a surface. As a result, a shell model potential for each ion connected via a spring [33-35] needs to be employed to handle the polarization of the oxygen ions. The total charge *q *of the ion is split between a core (of charge *q*-*Y*) and a shell (of charge *Y*), which are coupled by a spring constant *K *as follows:(2)

where δ_ij _is the core-shell distance. The potential parameters of the atoms of ZnO nanowire interactions adopted in the present study are listed in Table 1. The force field developed in this work is based on the interatomic potential derived by Oba et al. for ZnO, from which the potential parameters were fitted to the RS cubic structure [36]. These parameters have been demonstrated such that the structural and thermodynamic parameters including equilibrium volume, lattice constant, isothermal bulk modulus, and its pressure derivative at standard condition are in good agreement with available experimental data and the latest theoretical results [33,36-38]. Therefore, the potentials could increase the confidence level of this study.

**Table 1:** Parameters of Buckingham and shell model potentials used in simulation

	*A *(eV)	*ρ *(Å)	*C *(eVÅ^6^)	*K *(eVÅ^-2^)	*Y *(e)
Zn_c_-O_s_	700.3	0.3372	0.0		
O_s_-O_s_	22764.0	0.149	27.879		
O_c_-O_s_				74.92	-2.86902

## Results and discussion

**Figure 1:**
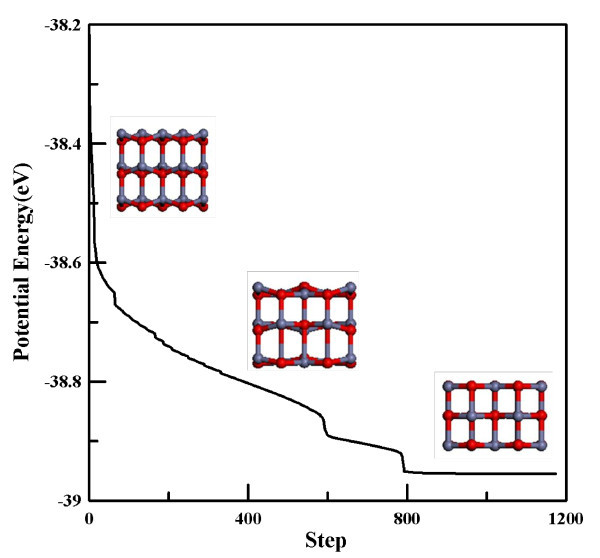
**Potential energy per ZnO monomer as a function of simulation iteration for structural minimization of Type I**. The insets are the corresponding structures.

**Figure 2:**
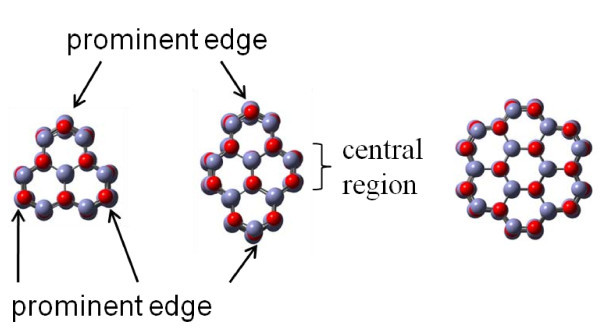
**Cross-section views of the structurally optimized ZnO**. (a)-(c) are the snapshots of ZnO nanowires for Type I-III.

This study addresses the tensile test of single crystalline HX phase ZnO nanowires of different wire width along the [0001] direction. Figure 1 shows the minimized procedure of potential energy per ZnO monomer as a function of minimization iteration, and the insets show the corresponding structure at different minimization steps. It is found that the energy gradually descends to -38.9 eV. The width of the nanowires becomes thicker and the WZ phase transforms to a HX phase after the energy minimization. The energy of the final equilibrium is slightly lower than that in the crystal phases (39.5-39.7 eV) [36,39] and is similar to that of ZnO in WZ, rocksalt, and blended structures confined within silica nanopores [39] and carbon nanotubes [40]. Note that the HX phase is an unbuckled WZ phase. This transformation phenomenon has been observed by the DFT study [25,26]. To understand the width effect on the mechanical property and the deformation mechanism in this work, three different widths of ZnO nanowires are optimized by energy minimization. Those optimized nanowire structures are denoted as Type I, II, and III. The cross-sectional structures of the ultrathin ZnO nanowires for Type I-III with diameters of approximately 0.7-1.1 nm are three-, two-, and sixfold axis symmetry structures, respectively. The cross-sectional views of the three optimized ZnO nanowires are presented in Figure 2, which are the most typical growth morphologies for ZnO nanowires found in experiments [41,42]. Compared to the average bond length of bulk ZnO with WZ structure, the value of Type I, II, and III are somewhat lower and are close to the DFT calculation results of 1.978, 1.989, and 1.999 Å [25], as shown in Table 2. Generally speaking, the ratio of the numbers of surface atoms to the total number of atoms increases as the width of the nanowire decreases. The relaxation of the surface atoms increases with the pre-compressive stress inside the nanowire [43]. Therefore, a nanowire width of smaller than critical size leads to an increase in the fraction of surface atoms significant enough to allow for the phase transformation to occur.

**Table 2:** Mechanical properties and bond length of ZnO nanowire with different type of structure under elongation test

	*Y *(GPa)	*σ_y _*(GPa)	*ε_y _*(%)	*L *(Å)
Nanowire (HX)				

Type I	697.919	47.516	0.113	1.973
Type II	687.401	47.617	0.108	2.008
Type III	592.880	39.033	0.094	2.018
Nanowire (HX) [25]				
Type I	537.6	-	-	2.068
Type II	532.6	-	-	2.071
Type III	303.5	-	-	2.079
Nanowire (WZ) [25]				
Type I	349.1	-	-	1.978
Type II	332.9	-	-	1.986
Type III	164.1	-	-	1.989
Nanobelt (WZ) [44]	339.76	36.332	0.046	-
	172.65	10.922	0.043	-
	140.37	8.625	0.02	-
Bulk [46]	144	0.2	-	-

**Figure 3:**
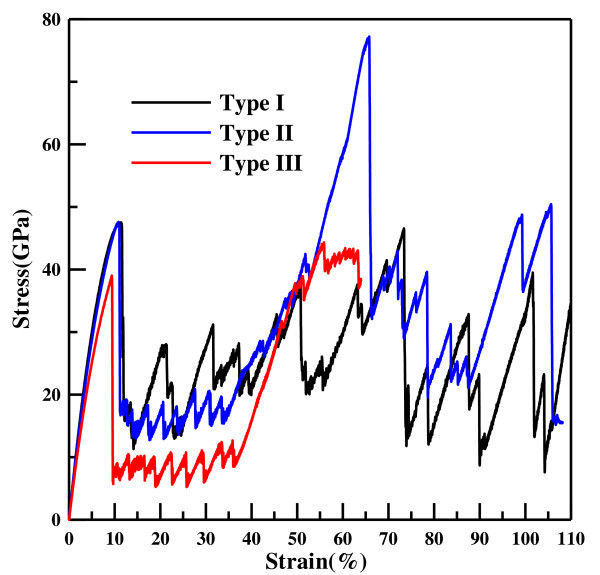
**Stress-strain relationship for Type I, II, and III**.

**Figure 4:**
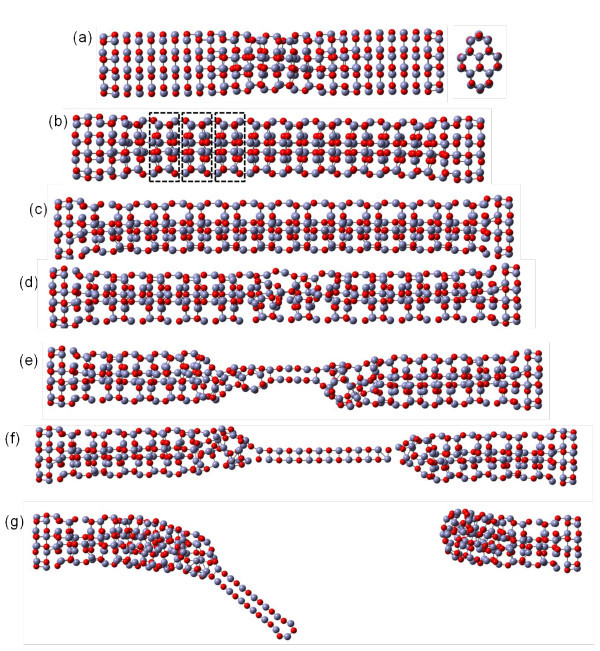
**Atomic configurations of Type II under uniaxial loading**. (a)-(g) show the corresponding snapshots of Type II at strain of 11.10%, 34.60%, 61.00%, 65.85%, 78.60%, 105.7%, and 108.18%.

For clearly discussing the detail of the deformation behavior, a central region and prominent edge are defined for Type I and II in Figure 2. The corresponding stress-strain profiles for the tensile process of different types of nanowires are shown in Figure 3. It is observed that as the wire width decreases, the maximum stress and the slope of stress-strain curve increase. Clearly, the both are much larger for Type I and II than for Type III. In the first stage, the stress increases linearly with slight fluctuation until the yielding occurs at yielding strain. The Young's modulus can be determined from the results of tensile test for the strain of 2%, using linear regression. The calculated results of Young's modulus for three types of nanowires are listed in Table 2, which corresponding to Wang et al.'s work [25] as listed in Table 2. This shows that the smaller the area of the cross-section, the greater the increase in the Young's modulus and the yielding stress. The variation tendency of the mechanical property as a function of width of ZnO nanowires has been verified by Kulkarni et al. [44] and Wang et al. [25]. At strain larger than the yielding strain, as shown in Figure 3, it is observed that the tendency of Type II and III are similar, both possessing two different stages II and III. At stage II, the stress-strain curve shows zigzag fluctuation from the yielding strain to the strain of approximately 35%. This phenomenon in Type II and III represents the local phase transformation, which is illustrated in Figure 4, which shows the side view of the Type II ZnO nanowire under the elongation process at different stages. It is observed that as the strain increases, the necking region of the HX structure gradually grows as shown in Figure 4a,b, because some of the ZnO bond parallel to the axis is broken, and the local HX structure becomes a buckled structure at the prominent edge of the cross-section of Type II. Here, we note that the structure is clearly buckled at the prominent edge of the cross-section of the nanowire, while it is slightly buckled at the central region. In addition, the phase transformation is generated symmetrically along the axis of nanowire, as can be seen by the rectangles in Figure 4b. The phase transformation of the ZnO nanowire has been observed in a loading and unloading process [24]. At stage III, the stress increases significantly with slight fluctuation, and is even higher than the yielding stress at the first stage. The slight fluctuation is due to the phase transformation near both ends, and the significant increase in stress is caused by the new phase as shown in Figure 4c. After the strain passes the maximum stress, the Zn-O bond is broken by a yielding stress of 80 GPa, as shown in Figure 3, and the corresponding snapshot is shown in Figure 4d. With a continuing increase in strain, the necking deformation gradually induces the nanowire to become a two-atom-wide chain in the middle region as shown in Figure 4e,f. After the strain of 108.18%, the two-atom-wide chain is fractured as shown in Figure 4g.

**Figure 5:**
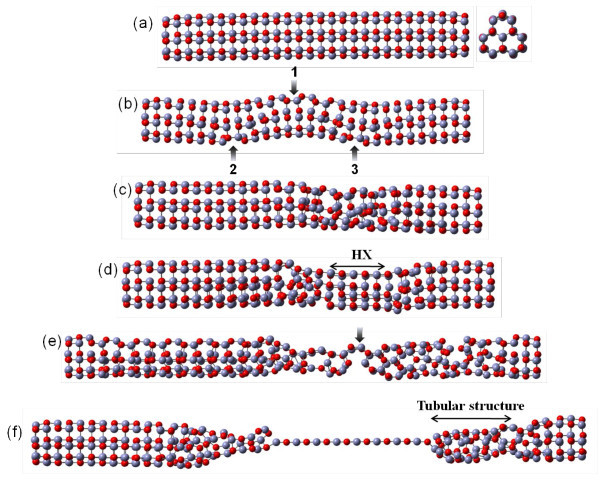
**Atomic configurations of Type I under uniaxial loading**. (a)-(f) show the corresponding snapshots of Type I at strain of 0.00%, 11.74%, 12.00%, 21.50%, 73.45%, and 100.00% respectively.

For Type I, the stress-strain curve is quite different than Type II and III. Figure 5 shows the deformation structure at different specific strains. Compared to Type II and III, the deformation behavior of Type I is totally different. As can be seen in Figure 5b, unlike the symmetrical phase transformation of Type II or III, the yielding of Type I is caused by a ZnO bond breaking, as shown by the arrow labeled 1. The unstable Zn and O atoms, then, lead to the first local buckling of the HX structure at the prominent edge of the cross-section of the Type I nanowire. Continuing, the local buckling of the HX structure induces the bending deformation of nanowire at the middle region and causes the second and third local buckling deformations as shown by arrows labeled 2 and 3. As the strain increases, the nucleation happens at the middle region (as illustrated in Figure 5c), and then the deformation region in the middle of the nanowire nucleates to a thinner HX structure (as illustrated in Figure 5d) until a strain of 21.5%. After the strain of 21.5%, the local HX structure gradually becomes a buckled structure. The buckled structure gradually extends to include the entire nanowire until a strain of 73.45%, as depicted in Figure 5e. Therefore, the stress-strain curve appears to show a significant zigzag fluctuation. After a strain of 73.45%, the middle region, marked by an arrow, starts to form the single atom chain. During the single atom chain growth, the buckled structure at both sides of the nanowire gradually relaxes and is restored to the original HX structure. The clear single atom chain bridged between two tubular tips is observed as shown in Figure 5f. Up until strain of 125%, the single atom chain is still not fractured.

**Figure 6:**
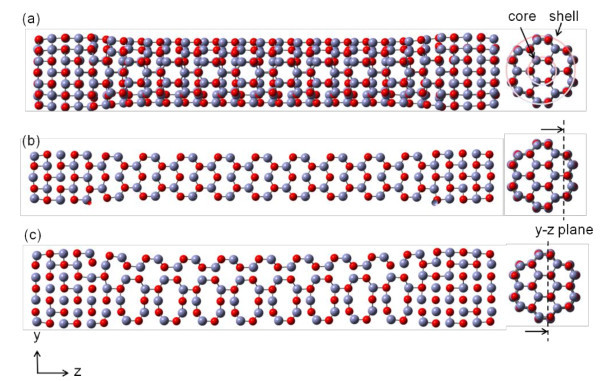
**Atomic configurations of Type I under uniaxial loading**. (a) and (b)-(c) show the corresponding snapshots and its cross-section side views of Type I at strain of 33.00%.

Comparing Types I-III, although the tendencies of the stress-strain curve of Type II and III are similar, the maximum strength of Type III is much lower than Type I and II. This is because the cross-section structure of Type III does not have any prominent edge, and therefore a lower stress. In addition, the phase transformation of Type III is generated on the whole ZnO nanowire uniformly as shown in Figure 6. This can clearly seen by the different cross-section side views of Type III in Figure 6b,c. As a result, tensile strength and Young's modulus of Type I and II are much higher than that of Type III.

We note that two stages of linear elastic deformation were observed in the tensile test of [0001]-oriented ZnO nanorods at a temperature higher than 300 K [27]. However, the simulation result in this work shows a three stage stress-strain curve, which could result from the very low temperature, leading to the slow growth of phase transformation in stage II. The super ductility of the single atom chain and two atom row structures of ZnO have been observed in [0001] ZnO nanowire under tensile loading by Dai et al. [29], as well as the carbon nanotube [45], and other metal nanowires [43]. In addition, Horlait and Coasne et al. [39,40] present the diversified atomic structure and morphology of ZnO nanostructure confined in carbon nanotube and porous silicas, discussing the effect of pore size and degree of pore filling on the self-assembly structure. The single atom chain, tubular structure, and both a four-atom ring and a six-atom ring are observed. These works verify the possible structural formation in this work.

## Conclusion

Molecular dynamics simulations of tensile tests of ultrathin ZnO nanowires have been employed to study intrinsic behavior. Three different types of ultrathin ZnO nanowires, with diameter from 0.7 to 1.1 nm, are simulated, with maximum tensile strength, yielding strain, and Young's modulus calculated. Simulated nanowires were of three different cross-sectional shapes, Type I, II, and III. As the width of the nanowire decreases, the yielding strength, yielding strain, and Young's modulus increase, while the bond length decreases. The yielding strength and Young's modulus of Type III is much lower than the other two types, because Type I and II have the prominent edges on the cross-section structure of the nanowire, which leads a stronger surface tension. Observation of the deformation mechanism shows that the HX structure of the ultrathin nanowire under uniaxial tensile loading transforms to a buckled structure to relax the tensile stress until the structure is buckled throughout the nanowire. This phase transformation process is reversible, which implies that the process is an elastic stretching process. In addition, we found that a one-atom-wide and a two-atom-wide chain appear before the nanowires are broken for Type I and II, respectively.

## Abbreviations

DFT: density functional theory; HX: hexagonal; MD: molecular dynamics; PE-FET: piezoelectric field-effect transistors; VLS: vapor-liquid-solid; WZ: wurtzite; ZnO: zinc oxide.

## Competing interests

The authors declare that they have no competing interests.

## Authors' contributions

WJ and JG participated in the data interpretation and drafted the manuscript. SP conceived of the study, and participated in its design and coordination. MH and CH participated in the MD programing and performed the statistical analysis. All authors read and approved the final manuscript.
